# Revisiting the complex time-varying effect of non-pharmaceutical interventions on COVID-19 transmission in the United States

**DOI:** 10.3389/fpubh.2024.1343950

**Published:** 2024-02-21

**Authors:** Gonghua Wu, Wanfang Zhang, Wenjing Wu, Pengyu Wang, Zitong Huang, Yueqian Wu, Junxi Li, Wangjian Zhang, Zhicheng Du, Yuantao Hao

**Affiliations:** ^1^Department of Medical Statistics, School of Public Health & Center for Health Information Research & Sun Yat-sen Global Health Institute, Sun Yat-sen University, Guangzhou, China; ^2^Guangzhou Liwan District Center for Disease Prevention and Control, Guangzhou, China; ^3^Guangzhou Joint Research Center for Disease Surveillance and Risk Assessment, Sun Yat-sen University and Guangzhou Center for Disease Control and Prevention, Guangzhou, China; ^4^Peking University Center for Public Health and Epidemic Preparedness and Response, Beijing, China; ^5^Key Laboratory of Epidemiology of Major Diseases, Peking University, Ministry of Education, Beijing, China

**Keywords:** COVID-19, NPIs, Bayesian hierarchical model, vaccination, shrinkage prior

## Abstract

**Introduction:**

Although the global COVID-19 emergency ended, the real-world effects of multiple non-pharmaceutical interventions (NPIs) and the relative contribution of individual NPIs over time were poorly understood, limiting the mitigation of future potential epidemics.

**Methods:**

Based on four large-scale datasets including epidemic parameters, virus variants, vaccines, and meteorological factors across 51 states in the United States from August 2020 to July 2022, we established a Bayesian hierarchical model with a spike-and-slab prior to assessing the time-varying effect of NPIs and vaccination on mitigating COVID-19 transmission and identifying important NPIs in the context of different variants pandemic.

**Results:**

We found that (i) the empirical reduction in reproduction number attributable to integrated NPIs was 52.0% (95%CI: 44.4, 58.5%) by August and September 2020, whereas the reduction continuously decreased due to the relaxation of NPIs in following months; (ii) international travel restrictions, stay-at-home requirements, and restrictions on gathering size were important NPIs with the relative contribution higher than 12.5%; (iii) vaccination alone could not mitigate transmission when the fully vaccination coverage was less than 60%, but it could effectively synergize with NPIs; (iv) even with fully vaccination coverage >60%, combined use of NPIs and vaccination failed to reduce the reproduction number below 1 in many states by February 2022 because of elimination of above NPIs, following with a resurgence of COVID-19 after March 2022.

**Conclusion:**

Our results suggest that NPIs and vaccination had a high synergy effect and eliminating NPIs should consider their relative effectiveness, vaccination coverage, and emerging variants.

## 1 Introduction

In the past 3 years, the value of non-pharmaceutical interventions (NPIs) in controlling infectious disease transmission has been greatly recognized in the context of the COVID-19 global pandemic. Implementing a series of containment NPIs, including school closing, workplace closing, canceling public events, gathering size restrictions, closing public transport, stay-at-home requirements, internal movement restrictions, and international travel restrictions (1), was one of the few tactics for such a new infectious disease. Many studies have reported the distinguished effects of NPIs on COVID-19 across the world (2–4), while long-term implementation of stringent NPIs also occasioned unintended issues in the economic recession (5), social problems (6, 7), and mental health outcomes (8). It has raised debates on whether and when an intervention could be lifted or eliminated (9, 10), which heavily depends on a comprehensive understanding of the effectiveness of NPIs based on real-world evidence.

Although we have trudged through the thorny forest with the end of the global health emergency of COVID-19 (11), there is still a risk of COVID-19 resurgence due to virus mutations. The risk of emerging infectious diseases has been increasing since the 21^st^ century, with several viral infectious diseases being pandemic within the last 20 years (12), which makes it more important to effectively implement NPIs to mitigate the epidemic. The practice of controlling COVID-19 provides an opportunity to clarify the effectiveness of NPIs, however, our understanding of the real-world effects of NPIs on mitigating COVID-19 transmission is not enough. The effectiveness of NPIs could change over time due to different compositions of variants of concern, socioeconomic features, vaccination, and policy compliance (13–16). Most previous studies focused on the early stage of the pandemic with the SARS-CoV-2 origin virus and the Alpha variant being dominant (3, 17, 18), however, evidence on the effectiveness in successive waves was relatively few, especially for the period of Omicron variant epidemic. Some modeling studies have discussed the varying effect of NPIs due to Omicron (19, 20), but the clues were not strong enough because the assumed population features and transmission parameters would be different from the real world.

Further, how vaccines worked with NPIs to eliminate the epidemic was also poorly understood. Several previous studies have explored the potential of lifting NPIs as the vaccination coverage increased to a pre-set level via mathematical models (20–23). Many of these modeling studies have provided constructive suggestions on delaying the relaxation of NPIs, but the reliability of simulation evidence would be challenged by the uncertainty of real-world vaccine efficacy and the emergence of new variants (24, 25). However, only one study has investigated the real-world combined effect of NPIs and vaccination and revealed a time-varying interaction effect between NPIs and vaccination (16).

Last but not least, multiple NPIs are always implemented and effective at the same time, but most previous studies failed to take this mechanism into consideration, which would limit the practical value in guiding policy decisions. Some studies were restricted to a specific combination of NPIs (e.g., lockdown) or regarded multiple NPIs as a whole by using a single index (13, 26, 27), which makes it hard to decide which kind of NPI could be modified. Another part of the study investigated the individual effect of a single NPI by constructing separate models for each NPI, ignoring the confounding of other NPIs (9, 15, 17). Some studies constructed a single model to simultaneously estimate the individual effect of multiple NPIs (18), but they did not consider the collinearity due to the high correlation among NPIs. Ignoring the collinearity issue can make results far away from the real effectiveness, leading to much uncertainty as to which NPIs are appropriate. Nevertheless, there is still no evidence untangling the complex effect of multiple highly correlated NPIs on COVID-19 transmission.

To provide more detailed evidence on the real-world effects of multiple NPIs across waves and pandemic variants, we revisited the complex time-varying role of NPIs in controlling COVID-19 transmission in the United States (United States) through a two-stage modeling framework. We first estimated bimonthly effects of NPIs, vaccination, and their interaction in forms of percentage reductions from time-specific basic reproduction number ( $R_{0,t})$ to effective reproduction number ( $R_{t})$ at the state level through a Bayesian hierarchical model with a spike and slab prior. Then, we utilized meta-analysis to pool the state-specific results and obtain the overall impact of NPIs in the U.S. over time.

## 2 Materials and methods

### 2.1 Data sources

#### 2.1.1 Outcome

To obtain a proper interpretation of the effectiveness of NPIs in mitigating COVID-19 transmission, we used the effective reproduction number ( $R_{t}$ ) to quantify the change in person-to-person transmission (27). Daily new diagnosed cases for the 51 states in the U.S. from 1 August 2020 to 31 July 2022 were obtained from *The New York Times* COVID-19 data repository, based on reports from state and local health agencies (28). To be consistent with *Our World in Data* and previous studies (16, 29), we estimated the daily state-specific $R_{t}$ using the Kalman filter, a Bayesian method modeling the growth rate of COVID-19 infections (30).

To consider the variation of basic reproduction numbers over time due to various compositions of variants of concern, we also estimated time-specific basic reproduction numbers ( $R_{0,t}$ ) to represent the basic transmission ability of the virus without any government response to the epidemic. First, we collected basic reproduction numbers for the original SARS-CoV-2 virus and the expansion factors for Alpha, Beta, Gamma, and Delta variants compared with the original virus from the literature (Supplementary Table S1). Due to a lack of evidence on the Omicron variant, we obtained its reported expansion parameter compared to the Delta variant. Then, the basic reproduction number for each variant was indirectly calculated by multiplying the expansion parameter with the basic reproduction number of the original virus or Delta variant (16, 31). Then, we collected the SARS-CoV-2 sequence data from the *Global Initiative on Sharing All Influenza Data* (GISAID) (32), and calculated the biweekly proportion of sequences of the above six variants between August 2020 and July 2022. Finally, we calculated $R_{0,t}$ by taking a weighted average of the basic reproduction numbers and proportions of the variants abovementioned.

#### 2.1.2 Measure of NPIs

Consistent with previous studies, we used the stringency index of NPIs assembled by the Oxford COVID-19 Government Response Tracker (OxCGRT) to quantify the intensity of overall and individual NPIs implemented by the governments (1). It is a composite measure based on multiple response indicators reflecting more or less intense government responses to the epidemic. We mainly focused on containment policies, including school closing, workplace closing, canceling public events, gathering size restrictions, closing public transport, stay-at-home requirements, internal movement restrictions, and international travel restrictions. Each NPI had 3 to 4 levels of intensity and all of them were first normalized to a value between 0 and 100 by a flag variable with a higher score indicating a stricter response (1). We defined the stringency index as the arithmetic mean of these eight NPIs index, indicating the overall intensity of NPIs implemented by the government. For a better model fit, these indexes were divided by 100 to downscale to 0–1 in this study.

#### 2.1.3 Other covariates

The fully vaccination coverages, i.e., proportions of people who completed the initial COVID-19 vaccination protocol, for the 51 states in the United States were collected from *Our World in Data* (33). Fully vaccination coverage was defined as the proportion of people who received all doses prescribed by the initial vaccination protocol. We did not consider the various efficacies of different types of vaccines because there was no accepted evidence on efficacies for different vaccines as well as their temporal variation. Using results from other populations and times would both introduce unknown confounding. We also considered temperature as a potential factor that would impact the relationship between NPIs and COVID-19 transmission (16). Thus, Hourly temperature gridded data (a resolution of 0.10 degrees) were obtained from the ERA5-Land dataset in the Climate Data Store maintained by the European Center for Medium-range Weather Forecasts (34). We calculated the daily mean temperature for each state by averaging data at all hours a day across all grids in the state and divided it by 10 to estimate the effect of temperature with per-10 degrees.

### 2.2 Statistical modeling

#### 2.2.1 The main model

We used a two-stage modeling framework to assess the effect of NPIs and their relative contributions in the United States by pooling state-level effects via meta-analysis with the random effect model. For each state, we assumed the effects of NPIs and vaccination to be stable within 2 months and estimated them through a Bayesian hierarchical model. The outcome in states was the natural logarithm of the state-specific ratio of effective reproduction number ( $R_{t}^{s}$ ) to time-specific basic reproduction number ( $R_{0,t}^{s}$ ), namely $\log\left(\frac{R_{t}^{s}}{R_{0,t}^{s}}\right)$ , where *t* represents the daytime in bimester *b*. We built a hierarchical linear model as follows,


$$\log\left(\frac{R_{t}^{s}}{R_{0,t}^{s}}\right)\sim \mathrm{normal}\left(\mu_{t}^{s},0.5\right)\text{,}$$

(1) $$\mu_{t}^{s}=\alpha_{b}^{s}+\overset{8}{\underset{i=1}{\sum }}\theta_{i,b}^{s}N_{i,t}^{s}+\beta_{1,b}^{s}V_{t}^{s}+\overset{8}{\underset{i=1}{\sum }}\gamma_{i,b}^{s}N_{i,t}^{s}V_{t}^{s}+\beta_{2,b}^{s}T_{t}^{s}\text{,}$$

where $N_{i,t}^{s}$ is the intensity index of *i*^*t*h^ NPI among the above eight containment in states at day *t*. $V_{t}^{s}$ and $T_{t}^{s}$ represent corresponding fully vaccination coverage and temperature, respectively. $\theta_{i,b}^{s}$ , $\beta_{1,b}^{s}$ , and $\gamma_{i,b}^{s}$ are the coefficients of individual NPI, vaccination and their interaction.

#### 2.2.2 The shrinkage prior

The Bayesian hierarchical model with shrinkage priors is a compelling method to deal with high-dimensional and correlated structure of predictors (35). Many simulation and application studies have reported the robustness of the results in high-dimensional regressions by using shrinkage priors (36, 37). Depending on the number and forms of prior distribution settings for the coefficients, it could be roughly divided into discrete mixture shrinkage priors, such as the spike-and-slab prior, and global–local (GL) shrinkage priors, including the horseshoe prior and Dirichlet Laplace prior (37, 38). The mixture shrinkage priors are to set a discrete mixture of a normal distribution with high density around zero (the spike) and a normal distribution with a large shape parameter (38), whereas the global–local shrinkage priors are continuous mixture of normal densities, which used the global parameter controlling the overall shrinkage of all the coefficients toward zero and the local parameters modifying the coefficient-specific shrinkage (39).

The spike-and-slab prior is often considered as the “gold standard” for sparse Bayesian estimation (39), so that we utilized it to cope with the collinearity issue due to the high correlation among NPIs, which could shrink small coefficients toward zero and assign large coefficients to the slab (40). We set each $\theta_{i,b}^{s}$ or $\gamma_{i,b}^{s}$ with a spike-and-slab prior as follows. For the sake of brevity with no loss of generality, we removed the state superscripts and the prior distribution could be written as,


(2) $$\vartheta_{i,b}\sim \delta_{i,b}\mathrm{normal}\left(0,\sigma_{i,b,\mathrm{spike}}^{2}\right)+\left(1-\delta_{i,b}\right)\mathrm{normal}\left(0,\sigma_{i,b,\mathrm{slab}}^{2}\right)\text{,}$$

$\delta_{i,b}\sim \mathrm{beta}($ 1,1),

where $\vartheta_{i,b}$ is $\theta_{i,b}^{s}\;\mathrm{or}\;\gamma_{i,b}^{s}$ in states which was constrained to be negative. $\delta_{i,b}$ is the inclusion probability of whether the coefficient equals zero with a noninformative prior of beta( 1,1) indicating that each coefficient has a 50% probability to be zero. $\sigma_{i,b,\mathrm{spike}}^{2}$ and $\sigma_{i,b,\mathrm{slab}}^{2}$ are the variances of normal distributions in which $\sigma_{i,b,\mathrm{spike}}^{2}$ is much smaller than $\sigma_{i,b,\mathrm{slab}}^{2}$ to make the probability density function closely around zero.

We put a half student-t prior with 3 degrees of freedom for these two hyperparameters to simultaneously ensure positivity and convergence (39), that is, $\sigma_{i,b,\mathrm{spike}}^{2}\sim \mathrm{half}\;\mathrm{student}-t\left(3,0,0.05\right)$ and $\sigma_{i,b,\mathrm{slab}}^{2}\sim \mathrm{half}\;\mathrm{student}-t\left(3,0,0.5\right)$ . We had a normal prior over the coefficients $\beta_{1/2,b}^{s}\sim \mathrm{normal}$ (0,0.5) and a student-t prior with 3 degrees of freedom for the intercept $\alpha_{b}^{s}\sim \mathrm{student}-t\left(3,0,1\right)$ .

#### 2.2.3 Effectiveness and empirical effect of NPIs

To get the overall effect of integrated NPIs, we obtain the posterior distributions of the overall effect of eight integrated NPIs and their interaction with vaccination by summing the $\theta_{i,b}^{s}$ and $\gamma_{i,b}^{s}$ in each iteration, that is, $\theta_{\mathrm{inte},b}^{s}=\overset{8}{\underset{i=1}{\sum }}\theta_{i,b}^{s}$ and $\gamma_{\mathrm{inte},b}^{s}=\overset{8}{\underset{i=1}{\sum }}\gamma_{i,b}^{s}$ , respectively. Then, the relative effectiveness of NPIs and vaccination could be calculated by $1-\exp\left(\theta_{\mathrm{inte},b}^{s}\right)$ and $1-\exp\left(\beta_{1,b}^{s}\right)$ . The estimated empirical effects taking the NPIs intensity and fully vaccination coverage into consideration are $1-\exp\left(\theta_{\mathrm{inte},b}^{s}N\right)$ and $1-\exp\left(\beta_{1,b}^{s}V\right)$ , respectively, which correspond to the realistic meaning of the reduction in $R_{0,t}$ regarding $R_{t}$ , i.e., $1-\frac{R_{t}}{R_{0,t}}$ , so as to the interaction between NPIs and vaccination. The relative contribution of *i*^th^ NPI was given as $\theta_{i,b}^{s}/\overset{8}{\underset{i=1}{\sum }}\theta_{i,b}^{s}$ (41), indicating the importance weight for each NPI in the overall effect of integrated NPIs. We estimated the relative effectiveness and empirical effect of integrated NPIs and the relative contribution of each NPI for every bimester (two-month) to account for seasonality and the impact of changing composition of dominant variant of concern.

To assess the model robustness, we modified the hyper-parameters in the spike-and-slab prior. Specifically, we changed the shape parameters in the half-student-t distribution to 0.3 and 0.03 for inclusion and exclusion, respectively. And we also alter the prior for $\delta_{i,b}$ from beta( 1,1) to beta( 3,5) with the assumption that only three of eight NPIs worked in a bimester. We also changed the linear model to a generalized linear model assuming the outcome of $R_{t}$ following gamma distribution which was conducted in previous research (16). Finally, we used the horseshoe prior, one of the most commonly used global–local shrinkage priors, with a $\mathrm{half}-\mathrm{student}-t\left(3,0,0.5\right)$ for the global parameter, to evaluate the influence of various shrinkage priors.

All the analyses were conducted in the R software (version 4.3.2). The Bayesian hierarchical model was performed through Markov Chain Monte Carlo (MCMC) methods by calling the Stan software via the ‘cmdstanr’ package (42). We ran four parallel chains for 2,000 iterations with the first 1,000 iterations of warmup to obtain 4,000 posterior samples for each state and bimester. All plots were drawn using the ‘ggplot2’ package.

## 3 Results

**Figure 1:**
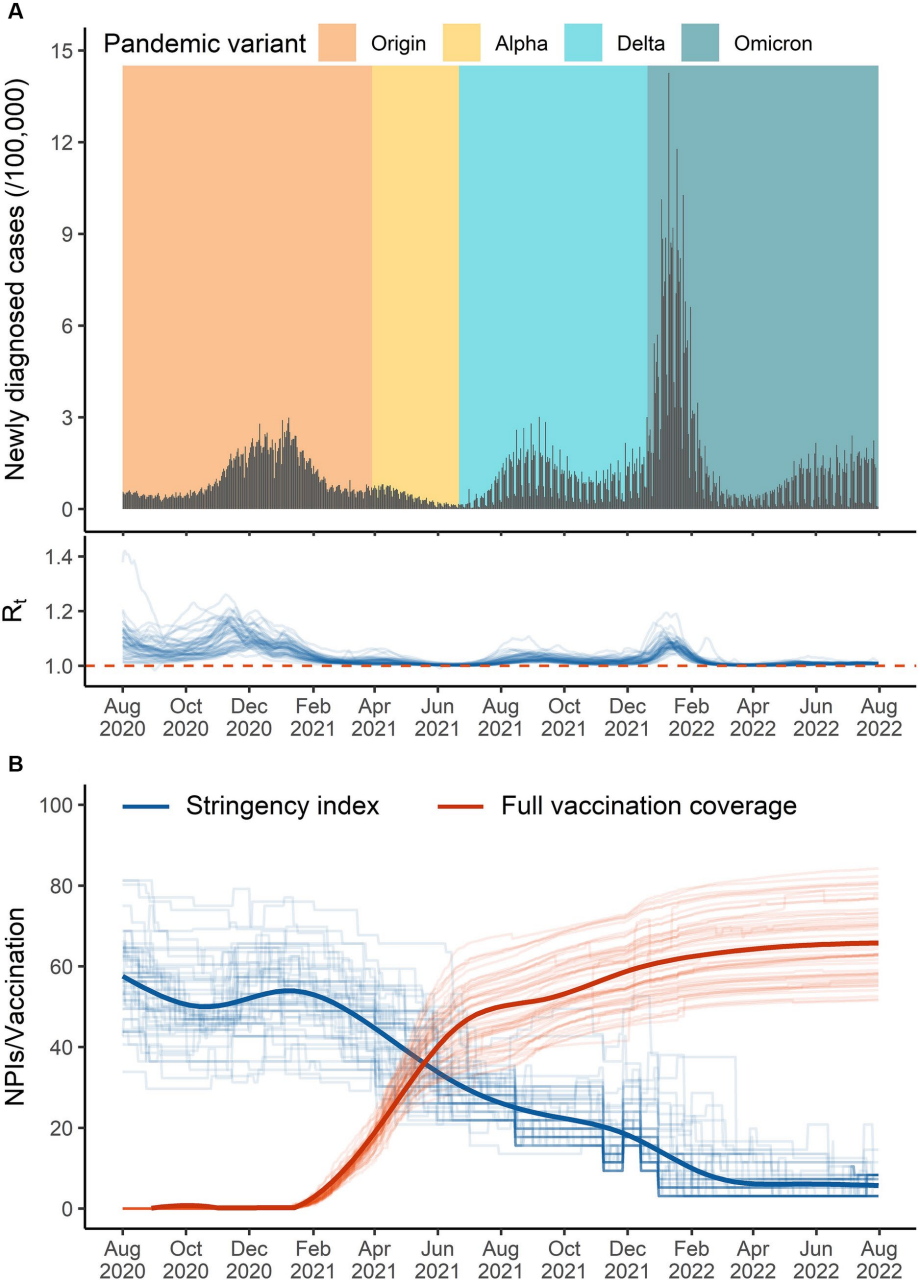
The overall temporal distribution of the data in the United States from 1 August 2020 to 31 July 2022. **(A)** Daily diagnosed cases (black bar), pandemic variant (colored background, defined as variants of concern that had the highest proportion), and effective reproduction number (blue lines in the bottom). **(B)** The stringency index (blue lines) and documented fully vaccination coverage (red lines) across 51 states (thick lines for the whole country).

During the study period, the variants of Alpha, Delta, and Omicron successively became dominant in April 2020, July 2021, and January 2022, respectively (Figure 1A). During August 2020 and February 2021, governments in the U.S. implemented NPIs with the stringency index being around 55. Since March 2021, governments continuously relaxed the intensity of NPIs and went to an “open” style in April 2022. Most of the pairwise correlations between individual NPIs were higher than 0.6, indicating a potential of collinearity should be considered (Supplementary Figure S2). The U.S. government commenced the mass vaccination campaigns at the end of December 2020. The fully vaccination coverage quickly climbed to 50% in August 2021 and slowly increased to 67.6% on 31 July 2022 (Figure 1B).

### 3.1 Estimated empirical effects of integrated NPIs

**Figure 2:**
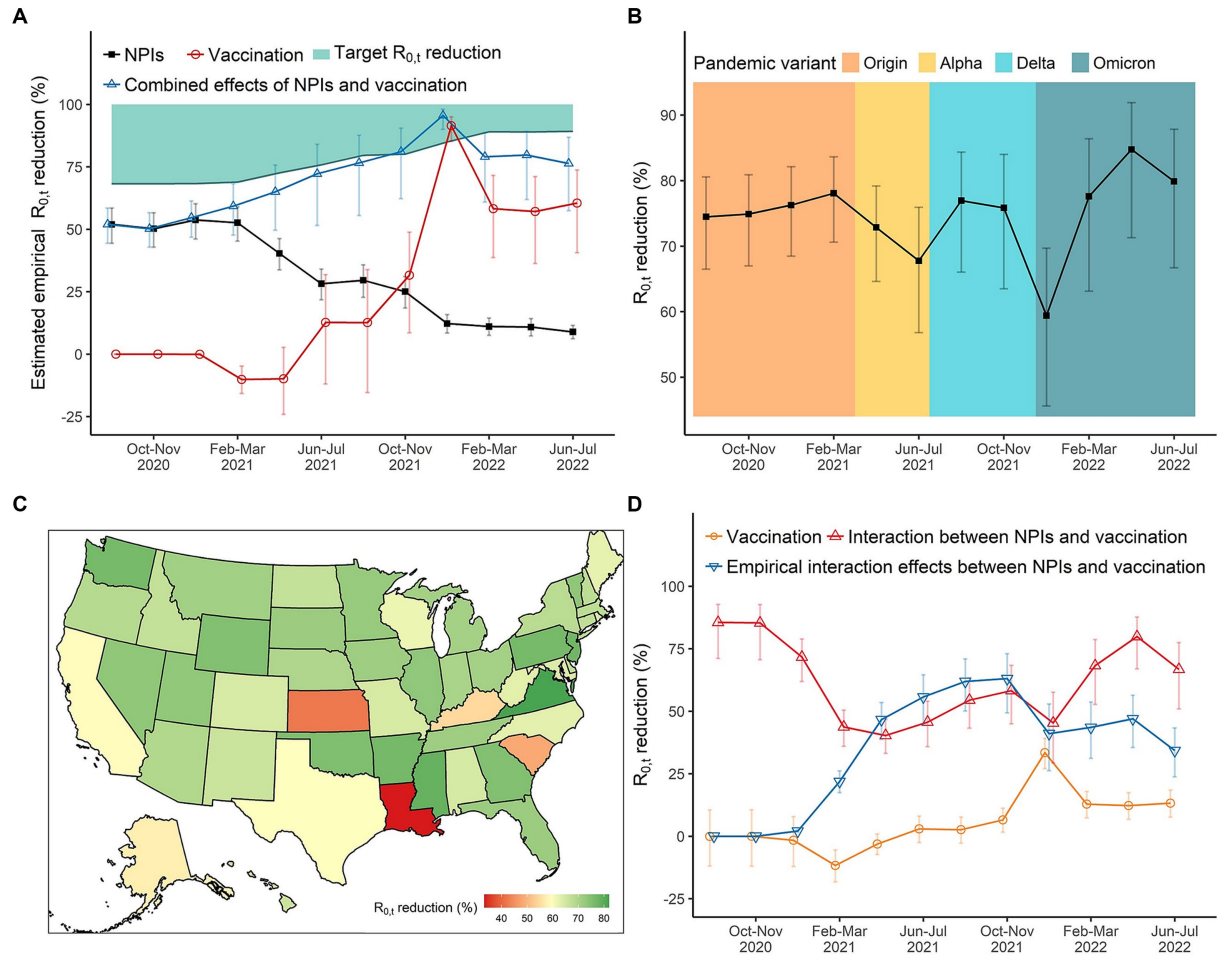
The effects of NPIs, vaccination, and their interaction on reducing COVID-19 transmission in the United States over time. **(A)** The empirical reductions in $R_{0,t}$ for integrated NPIs, vaccination, and their combined effects (integrated eight NPIs + vaccination + interaction). **(B)** The reductions in $R_{0,t}$ for integrated NPIs when all NPIs set at the highest level. We can compare the absolute effects of NPIs across periods of different pandemic variants. **(C)** The spatial distribution of integrated effects of NPIs during December 2021 and January in 2022 where the daily diagnosed cases reached the peak due to the Omicron variant. **(D)** The (empirical) effects of vaccination and its interaction with NPIs.

In August and September 2020, the estimated empirical reduction in $R_{0,t}$ attributable to integrated NPIs was 52.0% (95%CI: 44.4, 58.5%). The relative effect of integrated NPIs stabilized at around 50.2% ~ 53.7% until the intensity of NPIs decreased in April and May 2021 (Figure 2A). Thereafter, the empirical effect of integrated NPIs decreased to 8.8% ~ 12.2% during December 2021 and July 2022 no matter whether the fully vaccination coverage was continuously increasing. In the early stage of mass vaccination campaigns (February and March 2021), there was a negative effect of vaccination on reducing COVID-19 transmission, with reductions in $R_{0,t}$ of −10.1% (95%CI: −15.8, −4.8%), while its effect climbed to 91.5% (95%CI: 85.2, 95.1%) by January 2022, with the fully vaccination coverage reaching 60% in the U.S (Figure 2A). Due to the decreased effect of vaccination in the context of the Omicron pandemic, the overall empirical effect of integrated NPIs was not high enough to reach the target $R_{0,t}$ reductions in that period (Supplementary Figure S3).

### 3.2 Effect variations of integrated NPIs due to different pandemic variants of concern

In the original SARS-CoV-2 virus period, the effect of integrated NPIs was stable at about 74.5% ~ 78.1%. We found that the emergence of a new dominant variant of concern would decrease the effect of integrated NPIs. For instance, the effect decreased to 67.8% (95%CI: 56.8, 75.9%) in June–July 2021 as the Alpha variant became the dominant variant of concerns. And the effect decreased to 59.4% (95%CI: 45.6, 69.7%) in December 2021 and January 2022 with Omicron becoming dominant (Figure 2B). we observed that Louisiana, Kansas, and South Carolina had the lowest effects of integrated NPIs on $R_{0,t}$ reductions (Figure 2C).

### 3.3 Vaccination and its interaction with integrated NPIs

When the fully vaccination coverage was less than 60%, per-10% increase in fully vaccination coverage could merely reduce the $R_{0,t}$ . The effectiveness of vaccination increased to 33.4% (95%CI: 27.1, 39.2%) by January 2022 with the fully vaccination coverage exceeding 60%, but it reduced to 12.8% ~ 13.2% with the Omicron being dominant after February 2022 (Figure 2D). The interaction effect between vaccination and integrated NPIs was higher than 75% from August to November 2020. Although it decreased to around 40% in 2021, it was much higher than the main effect of vaccination (Figure 2D).

Taking the practical vaccination coverage into consideration, we found that the empirical effect of vaccination in the early stage of mass vaccination campaigns was also small, but the combined effects of integrated NPIs and vaccination in that stage were higher than their separate effects. From February 2021 to March 2022, syntheses of NPIs and vaccination were always enough to reach the target $R_{0,t}$ reductions (reducing the $R_{0,t}$ below 1) even the intensity of NPIs decreased. However, we saw the combined effect from April to July 2022 was not high enough, followed with a resurgence of COVID-19 after March 2022 (Figures 1A, 2A).

### 3.4 Relative contributions of individual NPIs

**Figure 3:**
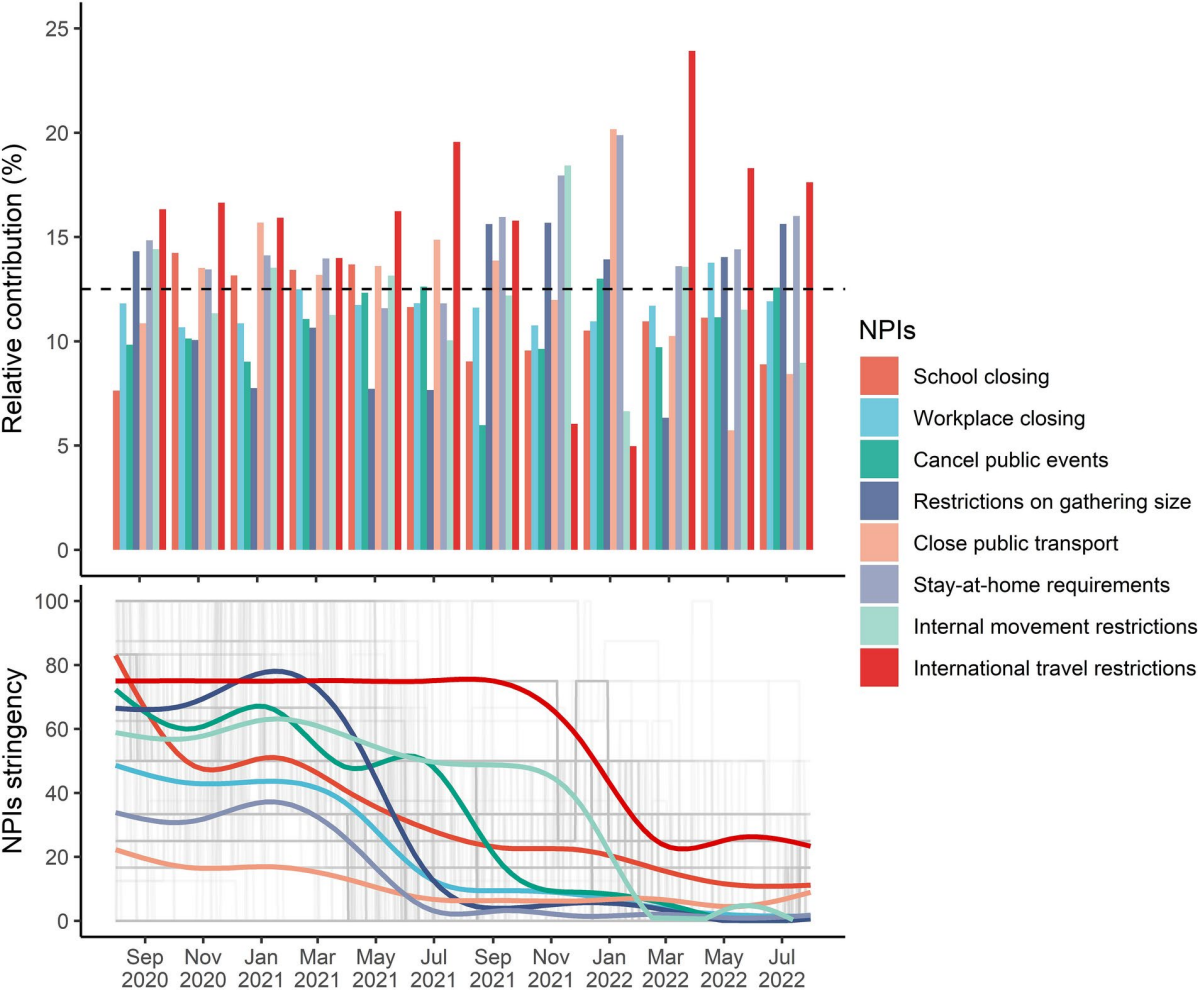
The bimonthly relative contribution of individual NPIs to the integrated effects.

The relative contributions of individual NPIs over time are shown in Figure 3. In almost time, international travel restrictions, stay-at-home requirements, and restrictions on gathering size contributed the most to the overall effects of integrated NPIs, with all corresponding relative contributions higher than 12.5%. However, the relative effect of international travel restrictions between October 2021 and January 2022 was low, with the Delta variant being dominant. The relative contribution of school closing was higher than 12.5% before the Delta and Omicron variants being pandemic, but it decreased to 8.9% ~ 11.1% after July 2021.

**Figure 4:**
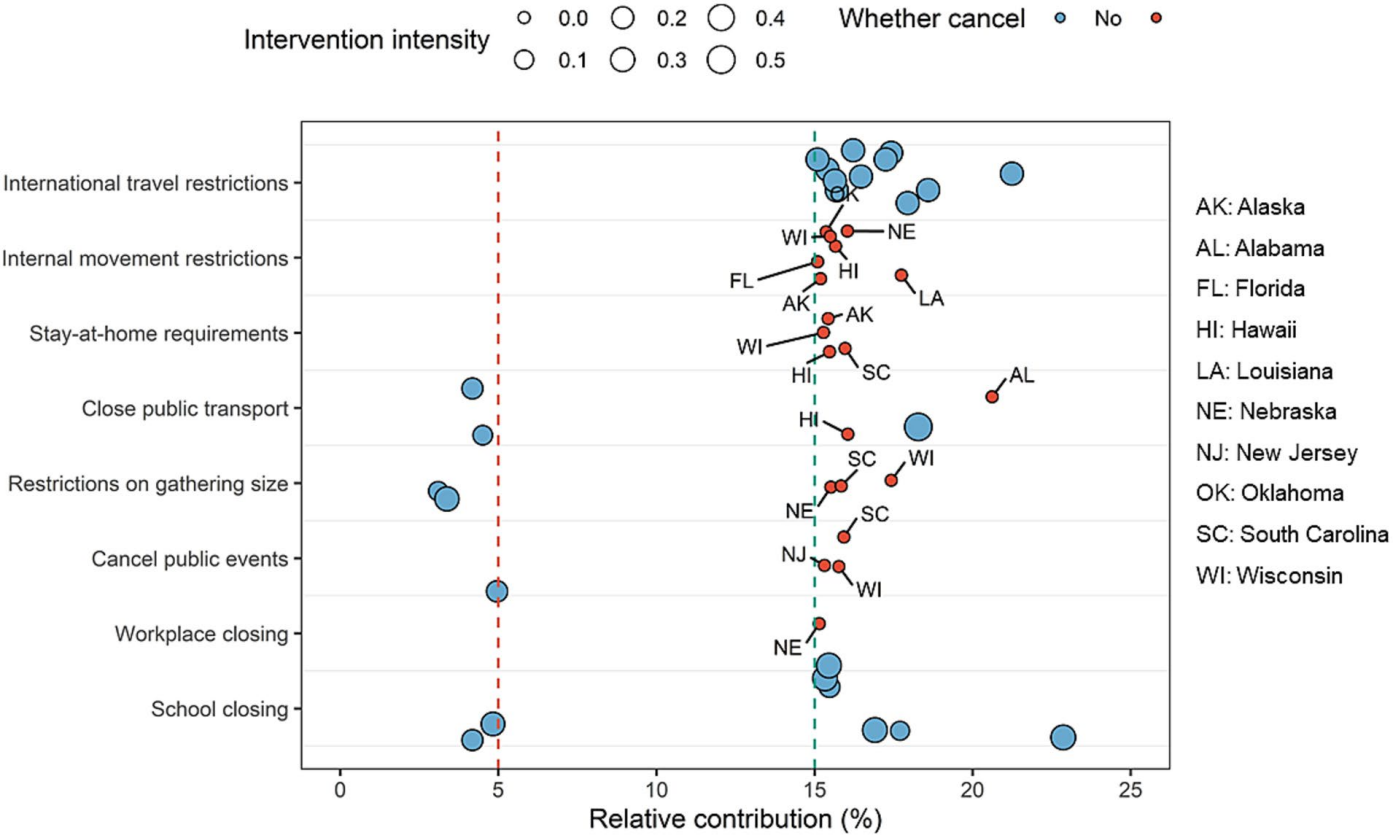
Selected NPIs with the lowest and highest relative contributions across 51 states in the United States during February and March 2022. Each bubble represents the relative contribution of one specific NPI in a state, with the size being proportional to the strength of NPIs. We considered that those NPIs with relative contributions higher than 15% should be maintained or strengthened, especially for internal movement restrictions, stay-at-home requirements, public transport closure, restrictions on gathering size, and public events cancelation in some states (red small bubbles).

The average intensity of individual NPIs continuously decreased below 20 after March 2022, but daily diagnosed cases started to increase in that period (Supplementary Figure S1) and implementation of NPIs synthesis was unable to reach the target $R_{0,t}$ reductions in the following months (Supplementary Figure S3). We found that the relative contributions of school closing in Nebraska and Idaho, public events cancelation in Hawaii, restrictions on gathering size in California and Hawaii, as well as closed public events in Nebraska and Wisconsin were below 5% (Figure 4). However, the relative contributions of internal movement restrictions in Alaska, Florida, Hawaii, Louisiana, Nebraska, Oklahoma, and Wisconsin, stay-at-home requirements in Alaska, Hawaii, South Carolina, and Wisconsin, close public transport in Alabama and Hawaii, restrictions on gathering size in South Carolina and Wisconsin, public events cancelation in New Jersey, South Carolina, and Wisconsin, and workplace closing in Nebraska were higher than 15%.

### 3.5 Sensitivity analysis

The distribution of R-hat statistics for all coefficients indicated that the main model in this study properly converged (Supplementary Figure S4). To assess the robustness of the model, sensitivity analyses were conducted by altering the hyper-parameters for the inclusion probability and variance of individual NPI coefficients. The results showed that the overall trends of the estimated effects of integrated NPIs, vaccination, and their interaction were consistent in all scenarios (Supplementary Figures S5–S7). The estimated empirical effects of NPIs and vaccination with the horseshoe prior were basically the same as the main results with the spike-and-slab prior in most bimesters, whereas the effect of NPIs in 2020 was lower when we used the horseshoe prior (Supplementary Figure S8).

## 4 Discussion

In this study, we constructed a Bayesian hierarchical model to estimate the real-world effects of integrated NPIs, vaccination, and the relative impacts of individual NPIs on reducing COVID-19 transmission among populations in the U.S. across multiple waves. We found that the effect of integrated NPIs alone continuously decreased since April 2021, along with the lifting of NPIs. The main effect of vaccination was low when the fully vaccination coverage below 60%, whereas it could still synergize with NPIs to effectively control the epidemic. International travel restrictions, stay-at-home requirements, and restrictions on gathering size were identified as the most important individual NPIs, and the elimination of these NPIs in many states would be responsible for the resurgence of the epidemic after April 2022. The proposed method in this study could provide a good tool to obtain more detailed clues to formulate cost-effectively comprehensive NPIs strategies in the future potential epidemic.

We found that the effectiveness of integrated NPIs was weakened in the short term when the Alpha and Omicron variants were dominant in the United States. There was no previous evidence on the real-world effectiveness variation of NPIs due to the emergence of new dominant variants of concern. This short-term decline in NPIs efficiency would be related to a higher transmission probability and lower policy adherence (31, 43, 44). The largest decline was observed in December 2021 and January 2022 when the daily diagnosed cases exceeded 0.6 million people (Figure 1A), closely following the epidemic caused by the Delta variant. Long-term implementation of NPIs and successive waves caused pandemic fatigue and reduced people’s adherence to policies (44, 45). More importantly, the results showed that the impact of NPIs was more correlated with the intensity of NPIs, with a decline in empirical effect along with the relaxation of NPIs (Figure 2A), which was consistent with previous estimation in Europe (16). And we observed a rebound in the COVID-19 epidemic after April 2022 with the intensity of NPIs decreasing to a very low level. It suggested that we should appropriately increase the intensity of NPIs when a new variant of concern becomes dominant.

Consistent with previous studies, we found vaccination alone could not mitigate the epidemic, especially in the early stage of mass vaccination campaigns. Many modeling studies have emphasized the necessity of maintaining the intensity of NPIs when the vaccination coverage is low (10, 16, 20). A more important finding in this study was that mass vaccination campaigns in the early stage could even increase the risk of transmission. Theoretically, mass vaccination campaigns could directly reduce individuals’ infection risk for vaccinated people and indirectly protect unvaccinated people by constructing herd immunity. However, the real-world efficacies of Pfizer-BioNTech and Moderna were lower than the results from clinical trials (46), and mass vaccination itself also increased the chance of contact and transmission due to large gatherings (47). Further, there is a threshold for fully vaccination coverage to achieve herd immunity, and it was estimated at least 60% of people in the U.S. needed to be fully vaccinated to obtain vaccine-derived herd immunity, which was consistent with the results in this study (48). Lack of herd immunity and large-scale gatherings were responsible for the increased risk of transmission for vaccination alone in the early period of mass vaccination campaigns, but fortunately, our results indicated that vaccination could synergize with NPIs and more attention should be paid to maintaining NPIs in that period.

Great heterogeneity of the effect rank of NPIs existed in previous studies (2). Ge et al. found that gathering restrictions played significant roles in controlling transmission across waves (15), but Liu et al. reported school closing as the most important NPI (18). The pairwise correlations of NPIs in this study indicated that a collinearity-related bias would exist in previous studies. There were very few studies considering this issue (49). In this study, we used a shrinkage prior in the Bayesian hierarchical model to solve this problem and estimated the relative contributions of individual NPIs. We found that international travel restrictions, stay-at-home requirements, and restrictions on gathering size were the most important NPIs in the U.S., and the relative contributions of individual NPIs would change over time.

According to the relative contributions of individual NPIs, we found that international travel restrictions, stay-at-home requirements, restrictions on gathering size, and cancelation of public events in some states should be strengthened after March 2022. There had long been calls to lift or eliminate NPIs because of the high socioeconomic costs and health trade-offs of NPIs, especially for school closing (50–52). We found school closing did not have an obvious impact on reductions in $R_{0,t}$ , which was lifted in almost states across the country. But more importantly, there was a lack of counterbalance of NPIs to reduce the $R_{0,t}$ below 1, following a resurgence of COVID-19. NPIs with high relative contributions, such as international travel restrictions, stay-at-home requirements, restrictions on gathering size, and cancelation of public events, might be eliminated too soon, especially in the states of Alaska, Hawaii, and Wisconsin. The results of this study suggested that mid-2022 was not the time to fully liberalize NPIs, and different states must lift individual NPIs according to their own conditions and should have corresponding hedging measures in other important NPIs.

By using a Bayesian hierarchical model with a shrinkage prior for highly correlated NPIs, this study provided elaborate evidence on the real-world effects of integrated and individual NPIs on mitigating COVID-19 transmission across time and populations. Some limitations in our analysis should be mentioned. First, due to lack of data availability, we used the biweekly reported proportions of sequences of main variants of concern to represent their actual proportions in the population. It introduced uncertainty in the effect estimation of NPIs and vaccination which should be interpreted with caution. Further, it was reported that demographics and comorbidities could also have great impact on the transmission of COVID-19 (53, 54), but there was not daily or monthly information on the state-level demographical status and comorbidity rate. More individual-level data were needed to investigate the role of these factors in COVID-19 transmission. Second, we were unable to consider the interactions among the eight NPIs in this study because of the unidentifiable interactions in such a sparse NPIs data which would also change over time. There was also no evidence on the interaction effects among NPIs, which should be further studied in the future. Third, we observed the threshold of fully vaccination coverage for herd immunity would be 60%, but more targeted analyses are needed to properly identify the threshold with consideration of variation in vaccine effectiveness across time.

In summary, we found that the overall impact of integrated NPIs on mitigating the COVID-19 pandemic was influenced by the new dominant variants of concern, especially for Omicron. The high correlation between intensity of NPIs and interaction with vaccination required us to simultaneously implement NPIs and vaccination when the fully vaccination coverage was low. School closing in some states could be lifted when the Omicron variant was dominant, but we should not eliminate internal movement restrictions and stay-at-home requirements in Alaska, Hawaii, and Wisconsin too soon to prevent the rebound of COVID-19.

## Data availability statement

Publicly available datasets were analyzed in this study. This data can be found at: https://github.com/DRGonghuaWu/USANPIspaper.

## Author contributions

GW: Data curation, Formal analysis, Investigation, Methodology, Writing – original draft, Writing – review & editing. WFZ: Formal analysis, Resources, Validation, Writing – review & editing. WW: Methodology, Validation, Visualization, Writing – review & editing. PW: Writing – review & editing. ZH: Writing – review & editing. YW: Writing – review & editing. JL: Writing – review & editing. WJZ: Conceptualization, Supervision, Validation, Writing – review & editing. ZD: Conceptualization, Funding acquisition, Resources, Supervision, Writing – review & editing. YH: Conceptualization, Funding acquisition, Resources, Supervision, Writing – review & editing.
