# Supplementary material for: Revisiting the complex time-varying effect of non-pharmaceutical interventions on COVID-19 transmission in the United States
Source: Front Public Health. 2024 Feb 21;12:1343950. doi: 10.3389/fpubh.2024.1343950 (PMC10915018; doi:10.3389/fpubh.2024.1343950)
Supplement: Supplementary file 1 [file Data_Sheet_1.docx]

**Supplementary materials for: Revisiting the complex time-varying effect of non-pharmaceutical interventions on COVID-19 transmission in the United States**

| Table S1 The basic reproduction numbers for different SARS-CoV-2 variants | | | | |
| --- | --- | --- | --- | --- |
| Variant | *R*_0_ | Lower | Upper | Paper |
| Origin | 3.15 | 2.86 | 3.45 | Yuqin Zhang et al. [1] |
| Alpha | 4.06 | 3.69 | 4.45 | Curran et al. [2] |
| Beta | 3.94 | 3.58 | 4.31 | Ge et al. [3] |
| Gamma | 4.35 | 3.95 | 4.76 | Campbell et al. [4] |
| Delta | 5.08 | 5.19 | 6.68 | Campbell et al. [4] |
| Omicron | 9.5 | 7.25 | 11.88 | Ying Liu et al. [5] |


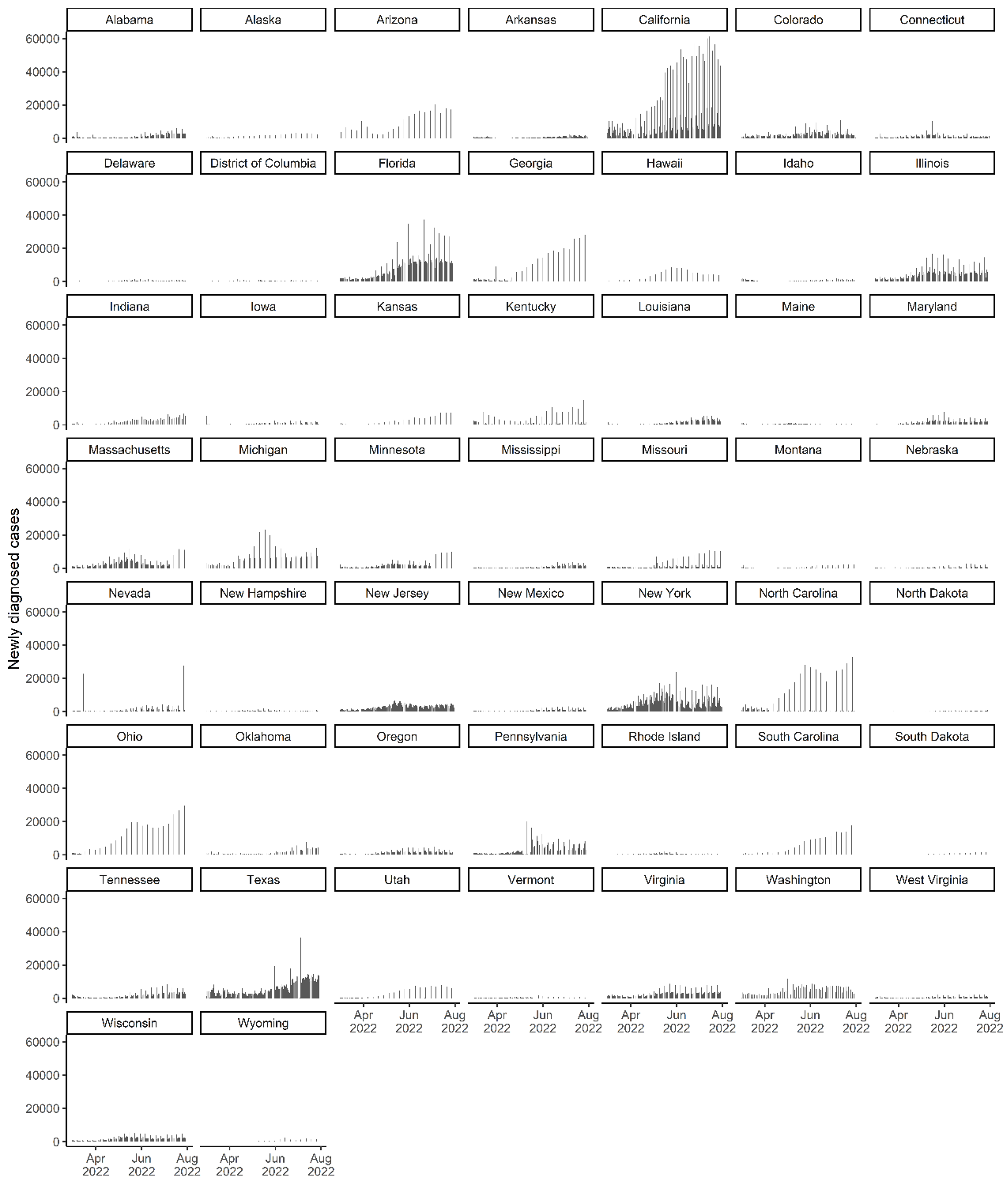


Figure S1 Daily diagnosed cases in 51 states after March 2022.


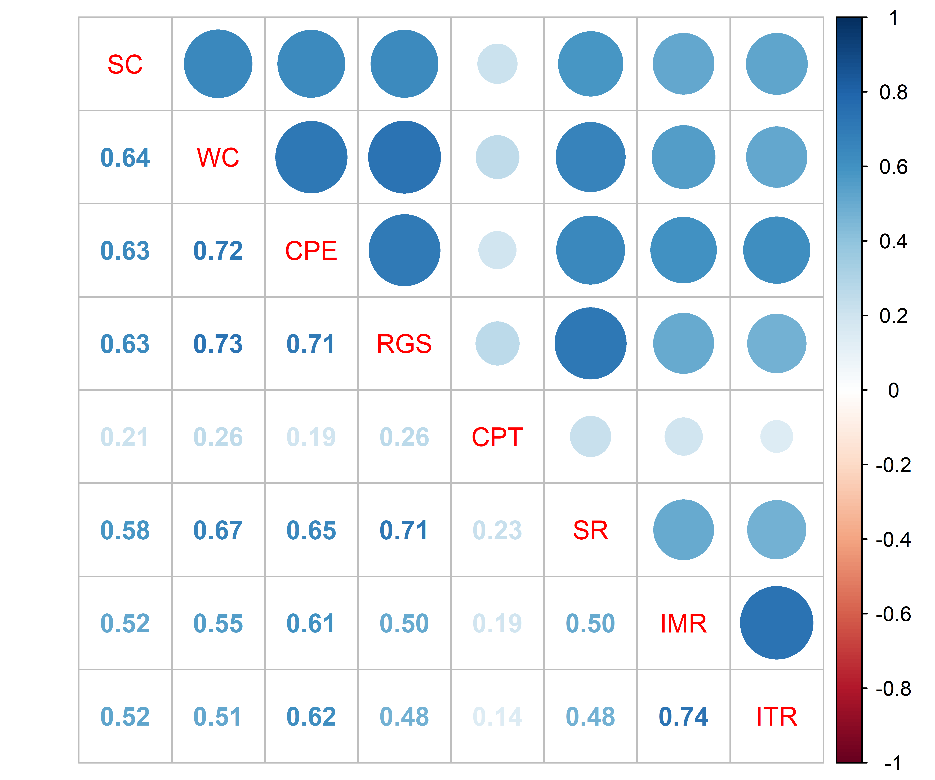


Figure S2 Pairwise correlation among eight NPIs in the U.S. between 1 August 2020


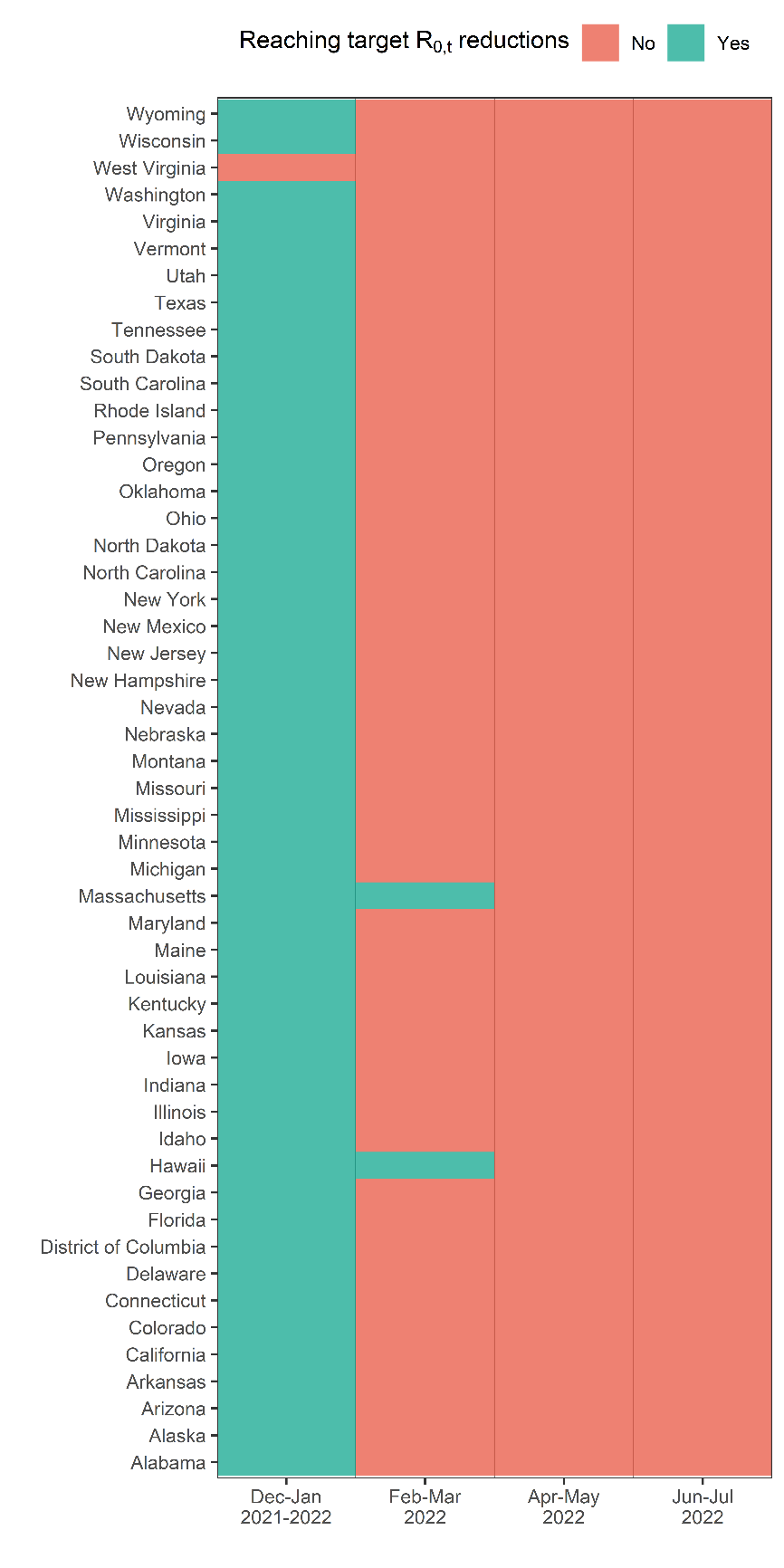


Figure S3 Heatmaps indicating that whether the NPIs intensity was high enough to reach the target reductions in $R_{0,t}$ in each state in the U.S. It was showed that almost all the states failed to reach the target value since February 2022.


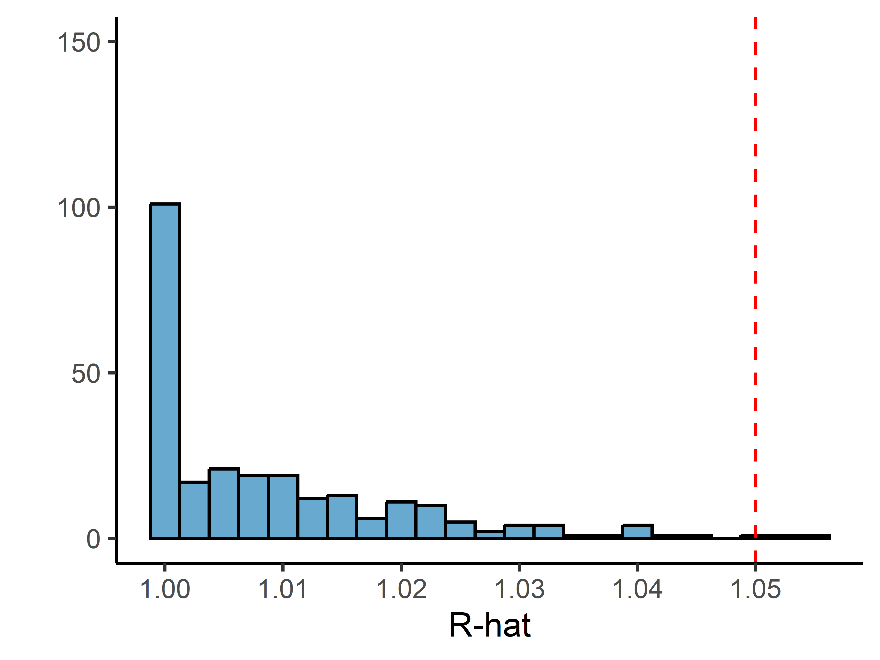


Figure S4 Distribution of R-hat for all coefficients in the model. It was showed that there was no R-hat exceeding the recommended threshold of 1.05, which indicated that the model in this study properly converged.


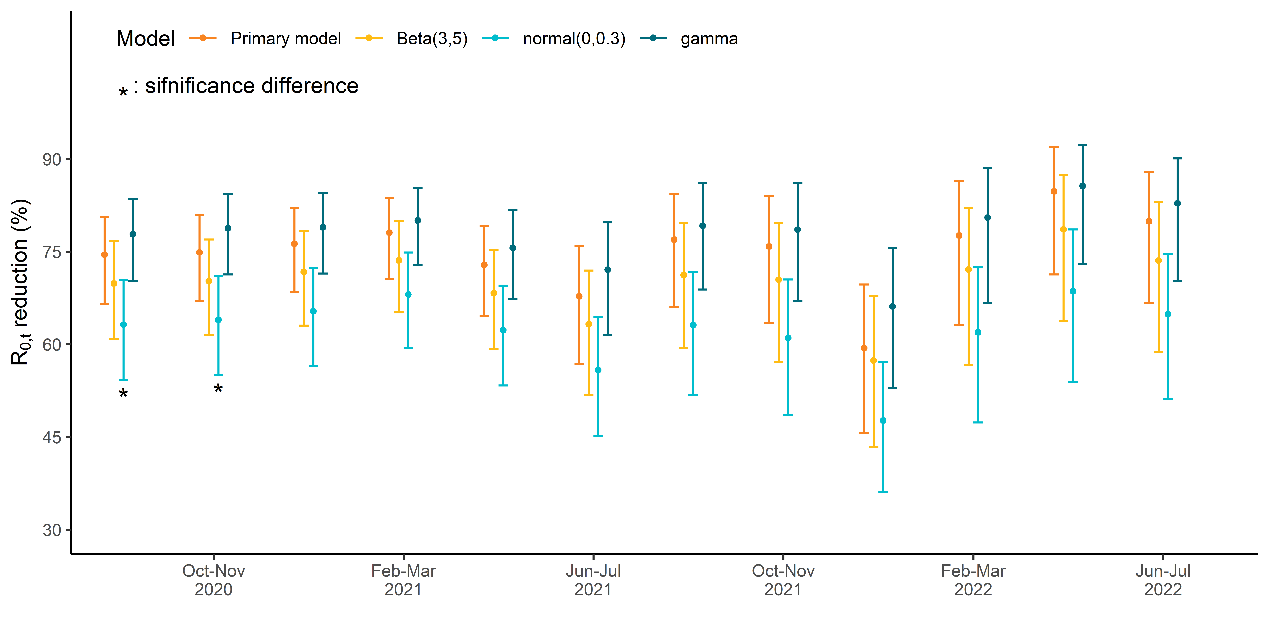


Figure S5 Effects of integrated NPIs on reducing COVID-19 transmission over time under different model settings.


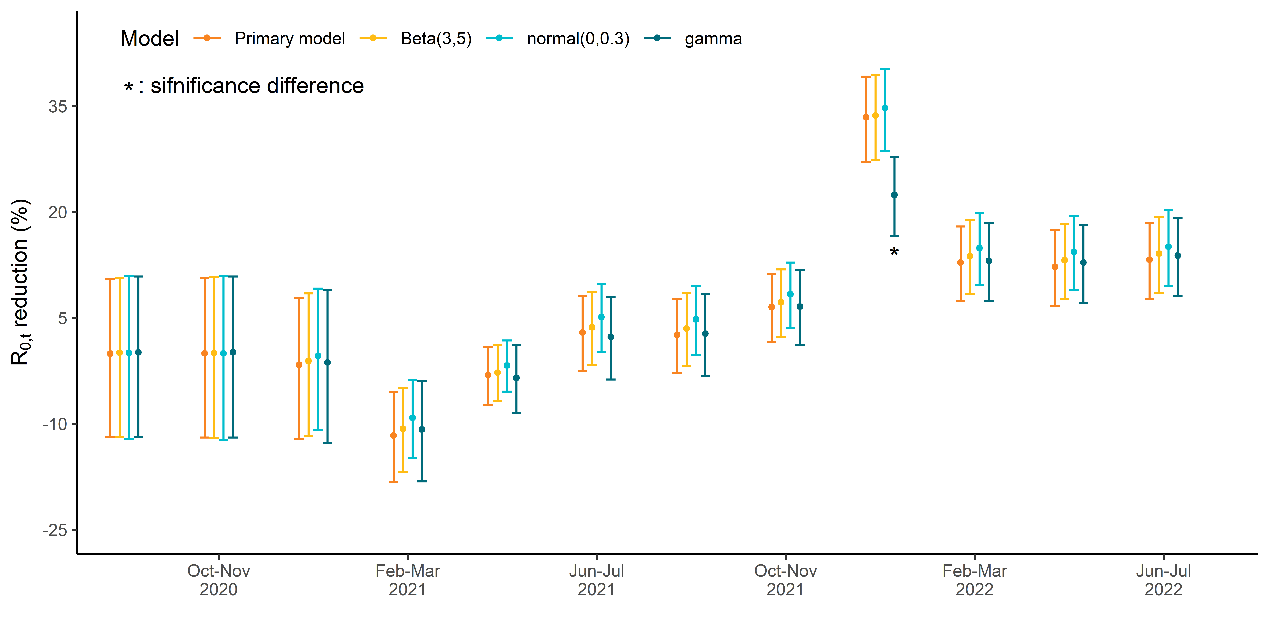


Figure S6 Effects of vaccination on reducing COVID-19 transmission over time under different model settings.


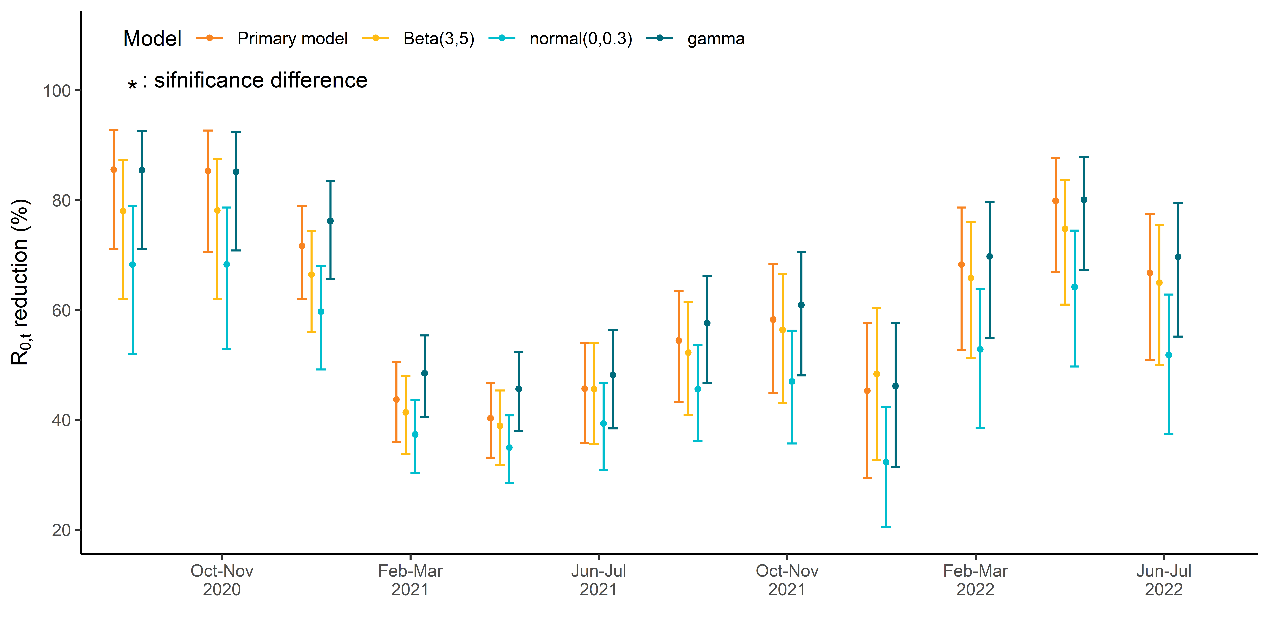


Figure S7 Effects of interaction between integrated NPIs and vaccination on reducing COVID-19 transmission over time under different model settings.


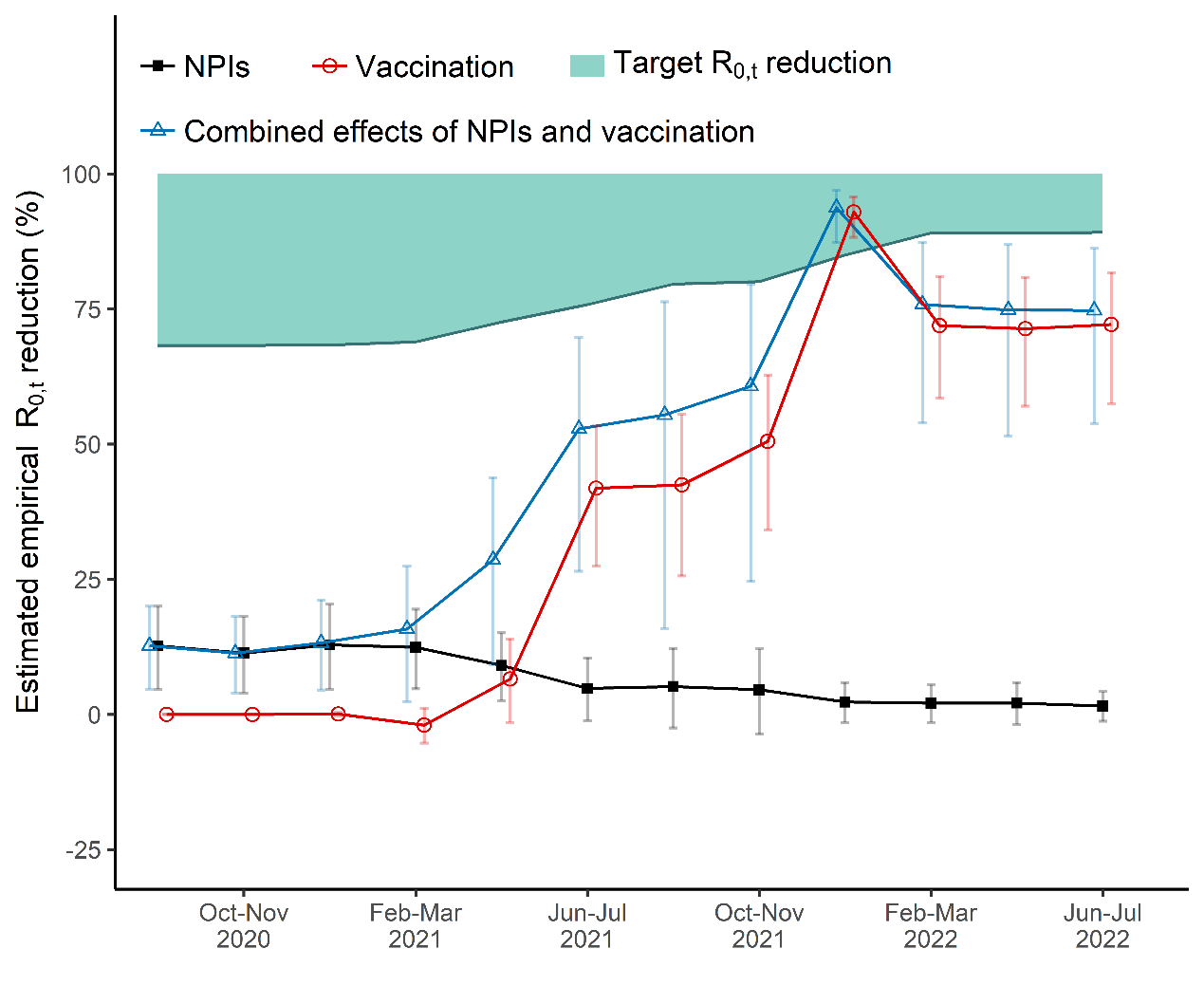


Figure S8 The estimated empirical effects of integrated NPIs and vaccination with the horseshoe prior

and 31 July 2022. SC: school closing, WC: workplace closing, CPE: closing public events, RGS: restrictions on gathering size, CPT: cancellation of public transport, SR: stay-at-home requirements, IMR: internal movement restrictions, ITR: international travel restrictions.


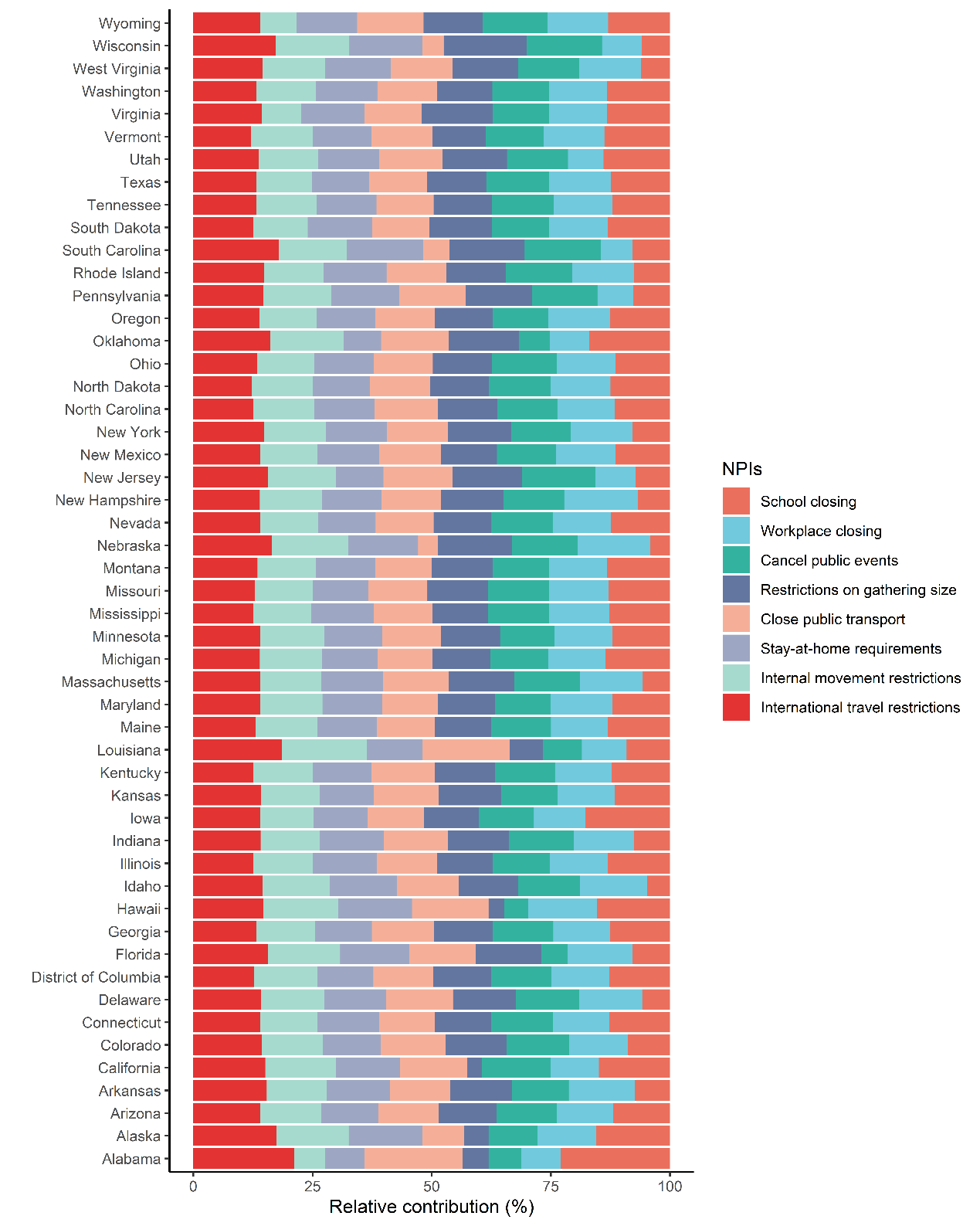


Figure S9 The relative contribution of individual NPIs across 51 states in the U.S. between 1 August 2020 and 31 July 2022.

**Reference**

1. Zhang Y, Wu G, Chen S, Ju X, Yimaer W, Zhang W, Lin S, Hao Y, Gu J, Li J: **A review on COVID-19 transmission, epidemiological features, prevention and vaccination**. *Medical Review* 2022, **2**(1):23-49.

2. Curran J, Dol J, Boulos L, Somerville M, McCulloch H: **Public Health and Health Systems Impacts of SARS-CoV-2 Variants of Concern: A Rapid Scoping Review**. In*.*: medRxiv; 2021.

3. Ge Y, Zhang WB, Wu X, Ruktanonchai CW, Liu H, Wang J, Song Y, Liu M, Yan W, Yang J *et al*: **Untangling the changing impact of non-pharmaceutical interventions and vaccination on European COVID-19 trajectories**. *Nat Commun* 2022, **13**(1):3106.

4. Campbell F, Archer B, Laurenson-Schafer H, Jinnai Y, Konings F, Batra N, Pavlin B, Vandemaele K, Van Kerkhove MD, Jombart T *et al*: **Increased transmissibility and global spread of SARS-CoV-2 variants of concern as at June 2021**. *Euro Surveill* 2021, **26**(24):2100509.

5. Liu Y, Rocklov J: **The effective reproductive number of the Omicron variant of SARS-CoV-2 is several times relative to Delta**. *J Travel Med* 2022, **29**(3).
